# Hospitalizations due to gastrointestinal Chagas disease: National registry

**DOI:** 10.1371/journal.pntd.0010796

**Published:** 2022-09-19

**Authors:** Ana Luiza Bierrenbach, Nayara Dornela Quintino, Carlos Henrique Valente Moreira, Renata Fiúza Damasceno, Maria do Carmo Pereira Nunes, Nayara Ragi Baldoni, Lea Campos de Oliveira da Silva, Ariela Mota Ferreira, Clareci Silva Cardoso, Desirée Sant’Ana Haikal, Ester Cerdeira Sabino, Antonio Luiz Pinho Ribeiro, Claudia Di Lorenzo Oliveira

**Affiliations:** 1 Hospital Sírio-Libanês, São Paulo, São Paulo, Brazil; 2 Universidade Federal de São João del-Rei, Divinópolis, Minas Gerais, Brazil; 3 Universidade de São Paulo, São Paulo, São Paulo, Brazil; 4 Universidade Estadual de Montes Claros, Montes Claros, Minas Gerais, Brazil; 5 Universidade Federal de Minas Gerais, Belo Horizonte, Minas Gerais, Brazil; FDA: US Food and Drug Administration, UNITED STATES

## Abstract

**Objectives:**

Analyze the hospitalizations of patients admitted for Chagas disease with gastro-intestinal involvement (CD-GI) in the Brazilian Unified Health System, describe the epidemiological profile, mortality and costs.

**Methods:**

This is an observational study that uses secondary data from the National Hospital Information System (SIH-SUS) for the years 2017–2019. CD-GI admissions were defined by specific ICD-10 codes that identify the main diagnosis.

**Results:**

From 2017 to 2019, there were 4,407 hospitalizations for CD-GI in Brazil, considering only public hospitals and those associated with the SUS. This corresponds to an average of 1,470 hospitalizations per year, or 0.6 per 100,000 inhabitants, with significant regional variation. Hospitalizations increased with age and were slightly higher in men. More than 60% were emergencies and in 50% the procedure performed was surgical. The most used code was the one for megaesophagus followed by megacolon. In-hospital mortality was 5.8% and 17.2% went to intensive care units. The median cost was USD$ 553.15 per hospitalization, and an overall cost of USD$ 812,579.98 per year to the SUS budget.

**Conclusion:**

The numbers, rates and costs presented here are possibly underestimated but they give us an idea of the overall profile of hospitalizations due to CD-GI, which are not rare and are related to significant in-hospital mortality. CD-GI is a neglected manifestation of a neglected disease.

## Author summary

The gastrointestinal involvement of Chagas disease is a neglected manifestation of a neglected disease. Despite this, it causes immense suffering to those affected, which lasts for years. The diagnosis is usually late and the treatment is difficult to access and inadequate, causing the patient to be operated on an emergency basis with major complications and, consequently, high mortality. Here we present the epidemiological profile and costs of public and government-funded hospitalizations, using the Brazilian national database.

## Introduction

Chagas disease (CD) represents a serious global public health problem nowadays, as it has long ceased to be restricted to Latin America due to international migration [1]. However, most of the 6 million to 7 million people infected are in Latin America, where vector transmission occurs. The vector is an insect (Triatominae, Hemiptera, Reduviidae) that carries the parasite *Trypanosoma cruzi*. Transmission also occurs by ingestion of contaminated food and from mother to child. Transmission via blood and derivatives is largely interrupted due to the care provided in transfusion centers. It is estimated that less than 10% of patients are diagnosed and less than 1% are treated [2].

The cardiac involvement of CD is the most common and the most studied and, therefore, will not be the focus of the current study [3]. Here, we intend to present the profile of hospitalizations resulting from involvement of the gastrointestinal tract (CD-GI), on which there is limited literature. Estimates in the literature on the percentage of patients chronically infected with this form of the disease are imprecise, with values ranging from 5 to 21%, which may reflect the lack of studies on the subject [2,4–6]. This involvement is so neglected that it does not even appear on the national list of preventable causes of death, even though the cardiac involvement appears as such [7]. However, it can lead to urgent hospitalizations and high-cost surgical procedures that could be avoided with early diagnosis and clinical follow-up. Better clinical management would certainly translate into improvement of the quality of life of these patients [8]. In order to study this, we will use the Hospital Information System (SIH-SUS) database, which has records of hospitalizations throughout the Brazilian territory in public hospitals and those contracted by the Unified Health System (SUS).

## Methods

This is an observational epidemiological study that uses national secondary data. The study uses individual SIH-SUS records from all over the country, collected and processed by the Brazilian Ministry of Health, extracted on December of 2020. The raw database is public and was downloaded from the Internet on the SUS Department of Informatics website [9]. Data indicating personal identification of hospitalized patients are not available in this database. SIH-SUS records the public hospitalizations as well as the private ones which are contracted by SUS. The data is used for reimbursement purposes. Although hospitals are supposed to register all admissions, in practice it is known that some do so only up to the monthly ceiling of the SUS reimbursement for each of them. The complete database contains around 12 million annual admissions, which account for approximately 75% of the total in the country [10,11]. Despite its eminently administrative / financial purpose, such data have been increasingly used in epidemiological studies.

A standardized admission form (AIH) is the initial data collection instrument, which is completed by doctors. AIHs contain the list of diagnoses that motivated hospitalization, coded with the International statistical classification of diseases and related health problems, 10th revision (ICD-10). Data are extracted from medical records at hospital discharge and recorded in the electronic system by trained clerks.

All SIH-SUS records referring to hospitalizations due to CD from 2017 to 2019, from all over the national territory were used. Subsequent hospitalizations were excluded as their numbers were greatly influenced by the Covid-19 pandemic. Patients who were not hospitalized and those who were in private hospital beds that were not contracted by SUS are absent from the data. It is important to note that there may be one or more hospitalizations of the same patient in the database, but as the data used had no personal identification, it was not possible to discriminate which records belonged to the same patient. Thus, the unit of analysis was the hospitalization record.

Hospitalizations identified as due to CD-GI were those with the codes, B57.3 Chronic CD with digestive system involvement, K23.1 Megaesophagus due to CD and K93.1 Megacolon due to CD in the variable “Main Diagnosis”. This will be the object of our main analysis.

However, there are hospitalizations in which these CD-GI defining codes were only located in the variables corresponding to “secondary diagnosis". To find out if the reason for these hospitalizations was due to CD-GI or if this disease was just a comorbidity, we manually reviewed all their codes for the "primary diagnosis" variable. Thus, records with a secondary diagnosis of CD-GI and main diagnosis of paralytic ileus, dysphagia or some condition or symptom compatible with CD-GI were classified as having this disease as the reason for hospitalization. On the other hand, records with a secondary diagnosis of CD-GI and main diagnosis with other Chagas codes without gastrointestinal involvement or other non-related conditions were classified as having this disease only as comorbidity. For patients who only had CD-GI codes listed as a secondary diagnosis, we present the codes listed as their primary diagnosis. We classified these cases into two categories: “CD-GI was the reason for hospitalization” or “CD-GI was a comorbidity”.

Apart from the number of hospitalizations due to CD-GI, we also studied the mortality of patients during hospitalization, the overall length of stay in the hospital, and if they went to the intensive care units (ICU). We also present the main surgical procedures performed with the patients during hospitalization, listed in a dedicated variable.

As for hospitalization costs, the values presented in the SIH-SUS were used for each patient. Costs variables are available in the database in both Brazilian reais and in US dollars, with the conversion rate corresponding to that of the day the data was entered into the system. These values, which are reimbursed to the hospitals by the government, are calculated by diagnosis/procedure groups, according to a standardized table used by SUS. In addition to direct medical costs (hospitalization, staff, use of diagnostic tests, therapeutic procedures and devices, materials and medicines), non-medical costs (hospital stays of a parent or caregiver) are also included.

We used medians and IQRs to summarize continuous variables and calculated frequencies and proportions for categorical variables. Summary statistics are presented by year, age group, sex, region, race, type of treatment (elective or urgent), bed specialty (surgical or clinical), mortality and intensive care unit (ICU) utilization. Hospitalization rates (per 100,000 population) were calculated by year, age group, sex, and region, using the population projections from the Brazilian Institute of Geography and Statistics (IBGE) available on the SUS Department of Informatics website [9]. Database management and descriptive analysis were performed using the Stata-17 software (Statacorp, College Station, Texas, USA).

The present study uses only public secondary data from databases administered by the Ministry of Health and openly available on official websites [9]. All data used were anonymous. Even so, researchers have taken every care to ensure data confidentiality. Ethical approval was not required.

## Results

During the years 2017, 2018 and 2019, there were 4,407 hospitalizations in the Brazilian SUS whose main diagnosis was CD with involvement of the gastrointestinal tract (CD-GI). There was no great variation from year to year, with an average of 1470 hospitalizations per year. Of the three ICD-10 codes used to define the disease, K23.1 referring to megaesophagus was the most common, representing more than half of the total (Table 1). For 85% of the records, the only available ICD-10 code was that of the main diagnosis, with no others in the variables dedicated to the secondary ones.

**Table 1:** Characteristics of hospitalizations due to Chagas disease with gastrointestinal involvement in the SUS. Brazil, 2017–2019.

Characteristics	N (%)
Total	4.407 (100.0)
Hospitalization year	
2017	1.504 (34.1)
2018	1.550 (35.2)
2019	1.357 (30.7)
ICD-10 code main diagnosis	
B57.3—Chagas disease (chronic) with digestive system involvement	621 (14.1)
K23.1—Megaesophagus due to Chagas’ disease	2.498 (56.7)
K93.1—Megacolon due to Chagas’ disease	1.288 (29.2)
Sex	
Male	2.287 (51.9)
Female	2.120 (48.1)
Age (years)	64.2 (52.1–73.8)
Age group (years)	
00–09	98 (2.2)
10–19	45 (1.0)
20–29	118 (2.7)
30–39	218 (4.9)
40–49	487 (11.1)
50–59	781 (17.7)
60–69	1.166 (26.5)
70–79	1.012 (23.0)
80 +	482 (11.0)
Race	
Brown	1.668 (37.8)
White	1.228 (27.9)
Unknow	1.146 (26.0)
Black	233 (5.3)
Asian	131 (3.0)
Indigenous	1 (0.0)
Region	
Southeast	1.929 (43.8)
Northeast	1.216 (27.6)
Central-West	892 (20.2)
South	258 (5.9)
North	112 (2.5)
Type of treatment	
Emergency	2.678 (60.8)
Elective	1.729 (39.2)
Specialties	
Surgical	2.301 (52.2)
Clinical	2.106 (47.8)
Death	
No	4.151 (94.2)
Yes in wards	148 (3.4)
Yes in intensive care units	108 (2.4)
Length of stay (days)	5.0 (3.0–9.0)
Intensive care unit utilization	
No	3649 (82.8)
Yes	758 (17.2)

Hospitalizations were slightly more frequent in men and there was a clear increase with age, except for the age of 0–9 years, which had a slightly higher frequency than that of 10–19 years. The median age was 64.2 years. Patients who self-identified as brown accounted for more than a third of hospitalizations, but it is important to note that just over a quarter of the race/color information was missing. Most hospitalizations were from residents of the Southeast and Northeast regions, followed by the Central-west. Over 60% of hospitalizations were classified as emergencies, i.e. when a patient seen in the emergency department is subsequently admitted to the hospital. The remaining 40% were classified as electives, i.e. when a doctor requests a bed to be reserved for a patient on a specific day. Analyzing the main procedure performed on the patient, 52.2% underwent some type of surgery and the 47.8% remaining underwent clinical treatment. Patients were hospitalized for a median of 5 days, with 17.2% using intensive care services. Overall, 256 (5.8%) patients died (Table 1). The mortality of patients who went to the ICU was higher than that of those who did not, although more patients died in wards than in ICUs. Of the 758 patients who went to the ICU, 108 (14.3%) died, and of the 3,649 patients who did not go to the ICU, 148 (4.1%) died.

For emergency hospitalizations, 62.7% performed clinical procedures and 37.3% surgical procedures. As for elective hospitalizations, 24.6% performed clinical procedures and 75.4% surgical procedures. As coded in the database, the most common surgical procedures are listed in Table 2.

**Table 2:** Surgical procedures performed on patients hospitalized due to Chagas disease with gastrointestinal involvement in the SUS. Brazil, 2017–2019.

Surgical procedures	N (%)
Surgical treatment of megaesophagus without resection / conservative	522 (22.8)
Surgical treatment of achalasia (cardiomyoplasty)	490 (21.4)
Multiple surgery treatment	376 (16.4)
Abdominal rectosigmoidectomy	311 (13.6)
Colostomy	266 (11.6)
Other procedures with sequential surgeries	138 (6.0)
Esophagogastrectomy	59 (2.6)
Partial collectomy (hemicolectomy)	19 (0.8)
All other	113 (4.8)
Total	2.294 (100.0)

**Fig 1:**
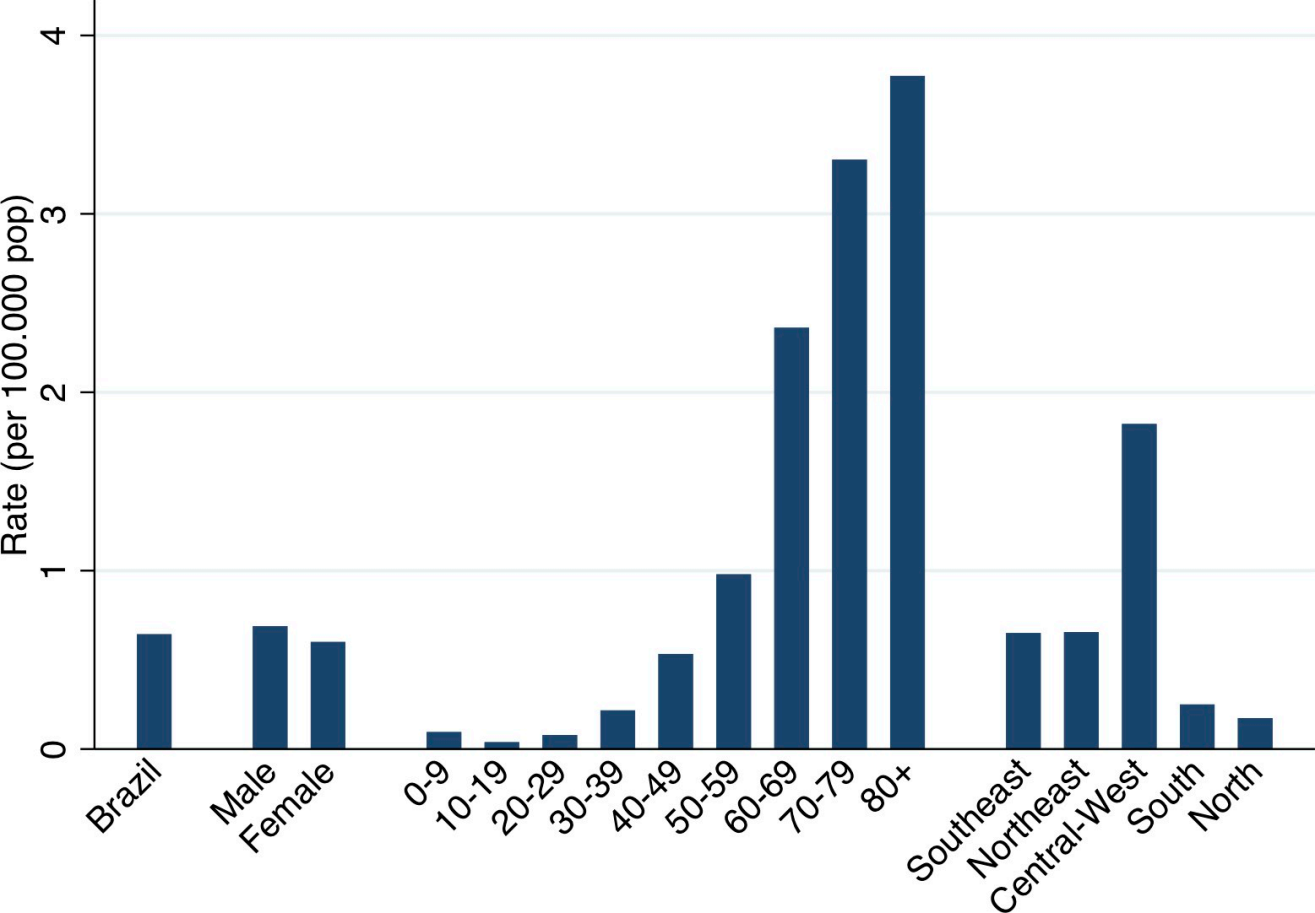
Rates of Chagas disease hospitalizations with gastrointestinal involvement in the Brazilian SUS stratified by sex, age group and region. Brazil, 2019.

In Fig 1, we present CD-GI hospitalization rates only for the year 2019. These rates were slightly higher for men and increased with age, similar to that found in the analysis of the numbers. However, rates in the Central-west region were higher than those in the Southeast and Northeast regions. The CD-GI hospitalization rate in 2019 in the Brazilian SUS was 0.6 per 100 thousand inhabitants (Fig 1). CD-GI hospitalization rates for the years 2017 and 2018 were similar to those of 2019.

**Fig 2:**
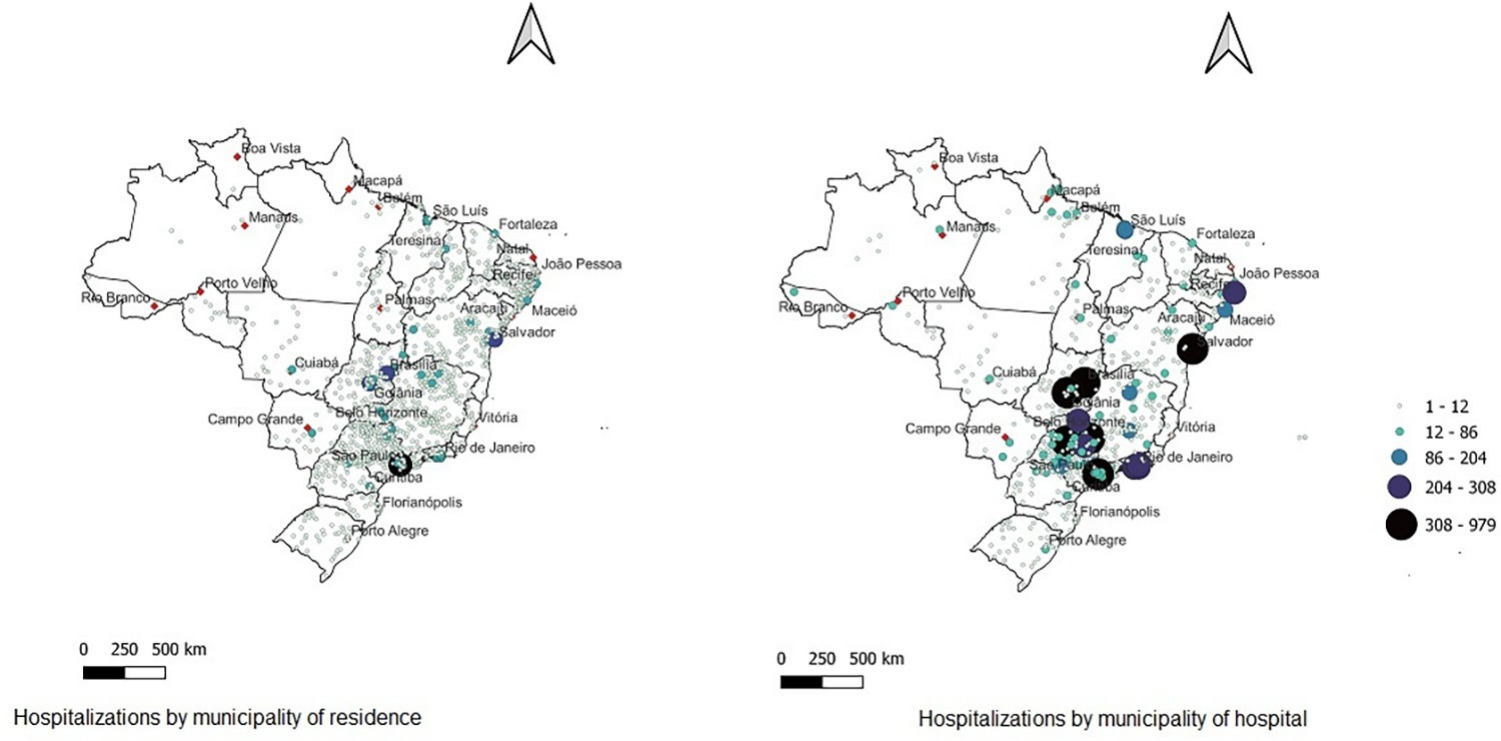
Spatial distribution of Chagas disease hospitalizations with gastrointestinal involvement in the Brazilian SUS by municipality of residence and of hospital. Brazil, 2017–2019. Note: Map from Instituto Brasileiro de Geografia e Estatística with data from Hospital Information System (SIH-SUS).

Patients hospitalized by CD-GI were residents in a total of 1,585 of the 5,571 Brazilian municipalities, although their hospitalizations occurred in only 582 ones. The spatial distribution of CD-GI hospitalizations by patient’s municipality of residence and of hospitalization can be seen in Fig 2. The maps show that patients reside throughout the country, with higher concentrations in capitals and some smaller cities at the confluence of the northeast, southeast and central-west regions. Surgeries are more concentrated in capitals and larger cities where complex medical care is more commonly available. The geographic distribution of the residences of patients under 18 years of age was similar to that of patients of all ages.

The 4,007 CD-GI hospitalizations in the years 2017–2019 had a median cost of USD$ 553.15 each, and an overall cost of USD$ 812,579.98 per year. The median costs varied by region, from USD$ 462.81 in the Center-West region to USD$ 614.22 in the South region. When the procedure performed was surgical, the cost was five times more than when it was clinical: USD$ 881.04 vs USD$ 194.91, respectively.

Table 3 shows the classification of the 660 hospitalizations in which the CD-GI codes were found only in the "secondary diagnosis" variables. Records were separated into two categories based on the code used in the main diagnosis variable. There were 335 (50.8%) records for which CD-GI was classified as a comorbidity, and there were another 305 (46.2%) for which CD-GI was classified as the main reason for the hospitalization. If we add these 305 to the 4,407 that we had already identified, the total number of admissions due to CD-GI would rise to 4,712, an increase of about 7%.

**Table 3:** Classifications of hospitalizations in which Chagas disease with gastrointestinal involvement codes were only located in the variables corresponding to the "secondary diagnosis".

ICD-10 code in the main diagnosis	N (%)
Gastro-intestinal Chagas Disease as the main reason for hospitalization	
K56—Paralytic ileus and intestinal obstruction	86 (28.2)
K59—Other functional bowel disorders	68 (22.3)
K22—Other esophageal diseases	43 (14.1)
R10—Abdominal and pelvic pain	20 (6.6)
K63—Other bowel diseases	16 (5.3)
K92—Other diseases of the digestive system	16 (5.3)
R13 –Dysphagia	10 (3.3)
K91—Intraoperative and postprocedural complications and disorders of digestive system, not elsewhere classified	9 (2.9)
K31—Other stomach and duodenal diseases	8 (2.6)
E46 –Unspecified protein-caloric malnutrition	7 (2.3)
K21—Gastroesophageal reflux disease	5 (1.6)
Z93—Artificial opening status (Colostomy,..)	4 (1.3)
E43—Severe protein-calorie malnutrition	3 (1.0)
K20 –Esophagitis	3 (1.0)
K93—Disorders of other digestive organs	3 (1.0)
K23—Esophageal disorders in COP diseases	2 (0.6)
E44—Protein-caloric malnutrition of moderate and mild degree	1 (0.3)
K30 –Dyspepsia	1 (0.3)
Partial total	305 (100.0)
Gastro-intestinal Chagas Disease as a comorbidity	
I50—Heart failure	30 (8.5)
J18—Pneumonia, unspecified organism	24 (6.8)
A41—Other sepsis	23 (6.5)
K92—Other disease of the digestive system	18 (5.1)
I64—Stroke not specified as hemorrhage or infarction	14 (3.9)
J15—Bacterial pneumonia, not elsewhere classified	13 (3.7)
C18—Malignant neoplasm of the colon	12 (3.4)
C15—Malignant neoplasm of the esophagus	11 (3.1)
B57—Chagas Disease*	10 (2.8)
N39—Other urinary tract disorders	9 (2.4)
A49—Bacterial infection from unspecified site	7 (2.0)
K55—Vascular disorders of the intestine	7 (2.0)
C16—Malignant neoplasm of stomach	6 (1.6)
Sum of other codes with smaller frequencies	171 (48.2)
Partial total	355 (100.0)
Total	660

* Cardiac involvement.

## Discussion

The numbers and rates of hospitalizations due to CD-GI presented here seem rather modest when compared to the universe of 36 million hospitalizations in public hospitals and in those contracted by the SUS in the same period. The interpretation of the results must take into account that CD-GI cases occur in the chronic form of the disease in a small proportion of infected people. Therefore, it can lead to hospitalization only in the most severe cases requiring surgical interventions or related to acute complications.

In addition, CD is typical of the most disadvantaged classes and of rural origin, which have less access to health services in general and hospitals in particular. Patients often have to make long journeys from rural areas to large urban centers in search of hospital care. This is shown in our results when we compare the number of municipalities of residence with the almost three times smaller number of municipalities of hospitalization.

It is also possible that some patients have been hospitalized outside the SUS, in private hospitals, although due to the precarious economic conditions of most patients with Chagas, this probably represents a minority of cases.

Another important aspect is that our numbers are likely to be underestimated even considering only hospitalizations within the SUS. Our results show that some hospitalizations had CD-GI codes listed only as a secondary diagnosis, with other codes related to symptoms or consequences of CD-GI appearing as the main diagnosis. Thus, it is fair to assume that there may be many other hospitalizations due to CD-GI in records that present these same codes, but that do not provide any information about their etiology and, consequently, were not identified by our methodology. The underestimation of hospitalizations for CD in the SUS was evidenced by studies that showed that hospitalization rates calculated using data from the SIH-SUS are sometimes lower than mortality rates for the same years calculated using data from the Mortality Information System [12].

In terms of costs, the overall values also seem small in relation to the magnitude of hospitalizations. This may be due to the fact that the average amount paid by the SUS as reimbursement to hospitals for each hospitalization is quite small and underestimated [13]. Regional cost differences are largely due to the unequal distribution of financial resources related to health services in the Brazilian territory [14]. Hospitalizations with surgical procedures were expected to incur higher costs, as observed. In a 2013 study that sought to estimate the economic burden of individuals in the chronic phase of CD, the annual cost of health care per infected individual ranged from USD$ 207–636 in Latin America, although it was much higher in the US, Canada and Australia, where it varied from USD$ 1,158–3,628 [15]. Although not fully comparable measures, the mean hospitalization value of USD$553.15 presented here falls within the range estimated in this study.

The clinical-demographic profile of CD-GI hospitalizations was similar to that shown in the literature for the prevalence of the disease, although there are only a few studies that have been dedicated to describing hospital morbidity with numbers greater than those of a series of cases [12,16,17]. Megaesophagus was the most frequently used code followed by that of megacolon, which are known to be the most frequently affected organs, although the involvement of other portions is known, but less frequent. [4–6,16]. Regrettably, there are no specific codes that identify the involvement of other portions of the GI tract in CD in ICD-10. In a classic study in which 1,708 autopsies were performed in chronic chagasic patients, 263 (15.4%) had mega viscera: megacolon was the most frequent finding, followed by megaesophagus and, thirdly, by the association of megacolon with megaesophagus [18]. Although megacolon may be more frequent than megaesophagus, it may not lead to as many hospitalizations or it may simply be more difficult to diagnose. The most common complaint of megaesophagus is dysphagia, which may occur earlier in the course of the disease or be more noticeable than the complaint of megacolon constipation, which, as a result, ends up being less investigated. Health professionals working in primary care are largely unaware of the need for screening and early diagnosis of chagasic patients, especially when their symptoms are common to other diseases [19].

The concomitance of two or more megaviscera, or gastrointestinal involvement together with cardiac involvement, could not be properly addressed in our study, since most of the records only provided information on the main diagnosis. Nevertheless, in the hospitalizations that had CD-GI codes listed only as a secondary diagnosis, we can see heart failure, stroke, and other codes in the main diagnosis variable, which may indicate the cardiac involvement of Chagas disease. Importantly, as there is an association between megaviscera and heart disease, part of the deaths could be due to a cardiovascular cause, precipitated by surgical stress. Codes for neoplasms of the intestine, esophagus and stomach are also listed. The association between CD-GI and esophageal and stomach cancer is well established in the literature, but the association between CD-GI and colon cancer has not been proved so far and continues to be studied [20–24].

In the chronic form, patients develop symptoms between the third and fifth decades of life, which worsen over time [4–6]. This is consistent with the finding that most of our patients were over fifty years old. Moreover, with the reduction of the vector transmission resulting from successful interventions in the 80s and 90s, due to a cohort effect, CD in Brazil became predominantly a disease of the elderly [25]. Although less frequently, we found hospitalized patients in younger age groups, as seen in other studies [26]. It is possible that, at least in part, these cases are due to vertical transmission [27]. Another possibility is that they occurred in the acute phase of the disease, when involvement of the GI tract can occur, although it is rare and transient [28].

The analysis of the racial distribution of cases hospitalized by CD-GI is complex because for over a quarter of them this information was ignored and it is likely that these values are not missing completely at random.[29] In general, what is expected is that this distribution reflects that of the population residing in the places most affected by the disease.

More than half of patients underwent some surgical procedure, which was not a big surprise. The literature indicates that there are several types of surgical treatment for both visceromegaly. For megacolon, surgery is predominantly considered in cases of refractory chronic constipation or in case of a major complication such as sigmoid volvulus or stercoral ulcer. The most common approaches rely on rectosigmoidectomy with retrocecal interpositioning, or with end-to-side low colorectal anastomosis [30,31]. As for megaesophagus, the surgical indication may occur earlier in the progression of the disease. Conservative alternatives include peroral endoscopic myotomy and lower esophageal sphincter pneumatic dilatation [32,33]. In addition, the surgical approach mainly includes laparoscopic Heller’s myotomy with partial fundoplication or esophagectomy. We believe that both endoscopic procedures and laparoscopic myotomy are represented in the surgical procedure code most used in these patients: "Surgical treatment of megaesophagus without resection/conservative". The second most common procedure, "Surgical treatment of achalasia (cardiomyoplasty)", refers to a procedure that is not performed endoscopically. It is important to point out that there must be endoscopic procedures performed in outpatient settings, which are consequently not included here.

Although not surprising, it is disheartening to find that almost 61% of hospitalizations were classified as emergencies, indicating that patients must have arrived at the emergency rooms with complications and/or great suffering that may, in turn, indicate poor or absent clinical follow-up. It may also indicate a lack of regulation of the hospital system in the country, in which the emergency room ends up being the gateway for many patients who cannot access otherwise [34]. Although most emergencies were resolved with clinical procedures, over one-third of them required surgery.

Our interest in studying which ICD-10 codes were used in the variable "main diagnosis" in patients whose CD-GI codes were located only in the variables "secondary diagnosis" was twofold. On the one hand, to discriminate for which of these patients the disease was the reason for hospitalization and, on the other hand, to eventually contribute to studies that assess the burden of morbidity and mortality by CD, listing some ICD-10 codes that can "hide", so to speak, hospitalizations or even mortality from this disease in large databases. In mortality studies, the use of multiple causes listed in all lines of the death certificate to better identify and understand deaths from a given cause is already well established [35]. We believe that in hospital morbidity studies, the same approach of broadly evaluating the list of all hospitalization diagnosis should also be applied. With the SIH-SUS data, this has become possible particularly since 2015, when the number of fields for the enumeration of each secondary diagnosis was expanded. However, it is still necessary to train and encourage the hospital registry staff to adequately fill in all the fields on the forms.

In summary, our study presents an overview of the hospitalizations attributable to CD-GI in the Brazilian SUS. It is a study that deals with the epidemiological and economic aspects of the public expenses due to this specific form of the disease. Numbers are possibly underestimated, but they give us an idea of the overall profile, which shows us that GI-CD is not rare and is related to significant in-hospital mortality. Monitoring burden and costs are crucial for the planning of interventions. Identifying the origin of hospitalized patients makes it possible to identify priority areas.
